# Controlling Product Selectivity in Oxidative Coupling of Methane by Identifying and Regulating Oxygen Species

**DOI:** 10.1021/acs.accounts.6c00102

**Published:** 2026-04-22

**Authors:** Jianshu Li, Vita A. Kondratenko, Kai Wu, Guiyuan Jiang, Evgenii V. Kondratenko

**Affiliations:** † State Key Laboratory of Heavy Oil Processing, China University of Petroleum, Beijing, Beijing 102249, P. R. China; ‡ Leibniz Institut für Katalyse e.V., Albert-Einstein-Str. 29a, 18059 Rostock, Germany; § Institute of Catalysis for Energy and Environment, Shenyang Normal University, Shenyang 110034, P. R. China; ∥ Shaanxi Key Laboratory of Low Metamorphic Coal Clean Utilization, School of Chemistry and Chemical Engineering, Yulin University, Yulin, Shaanxi 719000, P. R. China

## Abstract

The direct oxidation of methane, which is the main component of natural gas, shale gas, methane clathrates, and biogas, to value-added products is an economically attractive and environmentally friendly alternative to strongly endothermic methane steam reforming to synthesis gas (CO/H_2_). Among the different routes, the oxidative coupling of methane (OCM) to ethylene/ethane (C_2_-hydrocarbons) is the most promising one. A key limiting factor is insufficiently high selectivity to C_2_-hydrocarbons due to their overoxidation to carbon oxides (CO_*x*_) at industrially relevant degrees of methane conversion. Although it is generally agreed that both selective and unselective reactions are initiated by oxygen species on the surface of catalysts, the kind, role, and origin of these species remain elusive, which hampers the tailored design of catalysts.

In this Account, we summarize our recent progress in understanding how product selectivity in the OCM reaction can be tuned by controlling the type of oxygen species through catalyst composition or reaction conditions. The combination of in situ time- and temperature-resolved catalyst characterization with transient kinetic methods, i.e., temporal analysis of products (TAP) and steady-state isotopic transient kinetic analysis (SSITKA), has been proven to be effective for understanding the origin and role of oxygen species involved in selective and unselective pathways. We also present strategies for regulating the concentrations of selective and unselective oxygen species. For the Mn-M­(M = Na, K, Rb, or Cs)_2_WO_4_ system, the electronegativity of the alkali metal was found to influence the ability of the catalysts to form selective oxygen species from gas-phase oxygen. The binding strength of atomic oxygen species is a key parameter for hindering the oxidation of methane to CO_*x*_ over Gd_2_O_3_-based catalysts. This property can be adjusted by using a metal oxide promoter. The nature and concentration of different oxygen species can also be controlled through the use of steam or an alternative oxidizing agent, N_2_O, and by performing the OCM reaction in a chemical looping mode, i.e., by alternating between CH_4_- and air-containing feeds. Using steam in the latter option enabled us to largely enhance the productivity of C_2_-hydrocarbons, thus making this technology more attractive for large-scale applications. The knowledge summarized in this Account is expected to present insights for further studies in the development of selective catalysts for various alkane oxidation reactions and in the optimization of reactor operation.

## Graphical abstract

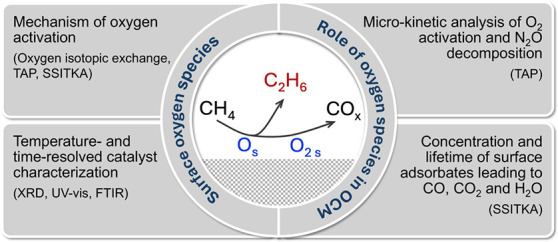

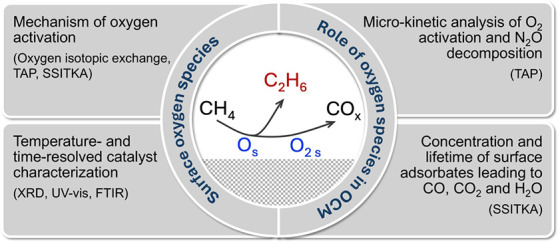

## Key References

-

Aydin, Z. ; Zanina, A. ; Kondratenko, V. A. ; Rabeah, J. ; Li, J. S. ; Chen, J. ; Li, Y. M. ; Jiang, G. Y. ; Lund, H. ; Bartling, S. ; Linke, D. ; Kondratenko, E. V. Effects of N_2_O and water on activity and selectivity in the oxidative coupling of methane over Mn-Na_2_WO_4_/SiO_2_: role of oxygen species. ACS Catal. 2022, 12­(2), 1298–1309 10.1021/acscatal.1c04915 .^1^ *The use of N*_*2*_ *O as an oxidant instead of O*_*2*_ *increases C*_*2*_ *-hydrocarbon selectivity. This is because N*_*2*_ *O has a low ability to generate diatomic oxygen species, which are involved in the formation of carbon dioxide, and to reoxidize the reduced catalyst. The latter effect lowers the coverage by both adsorbed and lattice oxygen species.*
-

Zanina, A. ; Kondratenko, V. A. ; Lund, H. ; Li, J. S. ; Chen, J. ; Li, Y. M. ; Jiang, G. Y. ; Kondratenko, E. V. The role of adsorbed and lattice oxygen species in product formation in the oxidative coupling of methane over M_2_WO_4_/SiO_2_ (M = Na, K, Rb, Cs). ACS Catal. 2022, 12­(24), 15361–15372 10.1021/acscatal.2c04916 .^2^ *Monoatomic adsorbed oxygen species formed from gas-phase O*_*2*_ *reveal higher reactivity and selectivity than lattice oxygen species of the catalysts. The electronegativity of the alkali metal in M­(Na, K, Rb or Cs)*_*2*_ *WO*_*4*_ *was identified as a descriptor governing the ability of the catalysts to form adsorbed oxygen species from O*_*2*_.
-

Wu, K. ; Zanina, A. ; Kondratenko, V. A. ; Xu, L. ; Li, J. ; Chen, J. ; Lund, H. ; Bartling, S. ; Li, Y. ; Jiang, G. ; Kondratenko, E. V. Fundamentals of unanticipated efficiency of Gd_2_O_3_-based catalysts in oxidative coupling of methane. Angew. Chem., Int. Ed. 2024, 63­(14), e202319192 10.1002/anie.202319192 38271543.^3^ *Gd*_*2*_ *O*_*3*_ *promoted with Ba, Na, or Sr was identified as a highly efficient catalyst for OCM with N*_*2*_ *O. The promoter influences the binding strength of monatomic oxygen species formed from N*_*2*_ *O on the surface of Gd*_*2*_ *O*_*3*_ *to hinder their recombination to unselective diatomic oxygen species, thus increasing the selectivity to C*_*2*_ *-hydrocarbons*.
-

Li, J. ; Chen, J. ; Zanina, A. ; Kondratenko, V. A. ; Lund, H. ; Jiang, W. ; Wu, K. ; Li, Y. ; Jiang, G. ; Kondratenko, E. V. Fundamentals of Li_2_CO_3_-Induced Enhancement of C_2_H_4_/C_2_H_6_ Selectivity in Oxidative Coupling of Methane over Mn-Na_2_WO_4_-Based Catalysts. ACS Catal. 2025, 15, 4770–4783 10.1021/acscatal.5c00492 .^4^ *The Li*_*2*_ *CO*_*3*_ *-mediated restructuring of the Mn–Na*_*2*_ *WO*_*4*_ */support catalysts is relevant to the generation of adsorbed oxygen species from gas-phase O*_*2*_ *. These material properties influence the lifetime and the concentration of surface intermediates, leading to CO and CO*_*2*_ *and accordingly the selectivity to C*_*2*_ *-hydrocarbons*.

## 1 Introduction

Selective-oxidation catalytic reactions form the basis of many large-scale processes producing the goods we need.^5–9^ They are also attractive in terms of energy consumption and current environmental requirements. Their role will remain crucial because oxygen is coproduced in water-splitting technologies for producing green hydrogen, enabling a sustainable economy.

Due to the widespread availability of methane, its oxidative conversion to the building blocks of the chemical industry, such as ethylene, synthesis gas, and methanol, could replace current energy-intensive technologies. For about 40 years, the oxidative coupling of methane (OCM) has been the most intensively investigated route to producing ethane/ethylene (C_2_-hydrocarbons) directly, yet it remains uncommercialized.^10–15^ This is due to the trade-off between methane conversion and C_2_-hydrocarbons selectivity (C_2_-selectivity), which has a great impact on the final price of these products.^16^ Controlling C_2_-selectivity poses the greatest challenge due to the complexity of the OCM reaction (Scheme 1). Given the decisive role of surface oxygen species in selective and side reactions, it is essential to understand which species are involved in these reactions and how they are formed from gas-phase oxygen (O_2_). These aspects have only recently received more attention and are the subject of controversy.

**Scheme 1:**
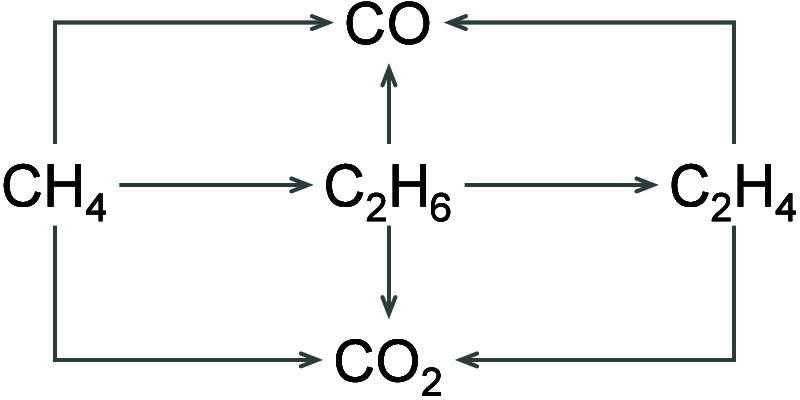
Overall Scheme of Product Formation in OCM

Herein, we highlight the potential and advantages of (micro)­kinetic analysis also using isotopic tracers, combined with time-resolved, spatially resolved, and temperature-resolved catalyst characterization for (i) uncovering the pathways leading to the target and side products, (ii) identifying the oxygen species involved in these reactions, and (iii) providing kinetic descriptors of product selectivity. Our approach enabled us to identify the strategies for regulating the concentration of selective and unselective oxygen species, which are relevant for catalyst design and optimizing reaction conditions. We also demonstrate our achievements in terms of C_2_-selectivity in comparison with state-of-the-art catalysts. Finally, we discuss the remaining challenges and provide our views on future developments.

## 2 Surface Oxygen Species and Methods for Their Identification

The formation of surface oxygen species from O_2_ on metal oxide catalysts typically occurs through (i) adsorption, (ii) electron transfer(s) from the catalyst to the adsorbed oxygen, (iii) dissociation of the charged oxygen, and (iv) incorporation into the lattice (Figure 1a). Both diatomic (O–O)_ads_ and monatomic (O)_ads_ adsorbed oxygen species can be formed. Various techniques are applied to identify the type of surface oxygen species and can be classified into (i) in situ/operando spectroscopic methods, (ii) ex situ measurements, and (iii) transient kinetic analysis (Figure 1b).

**Figure 1:**
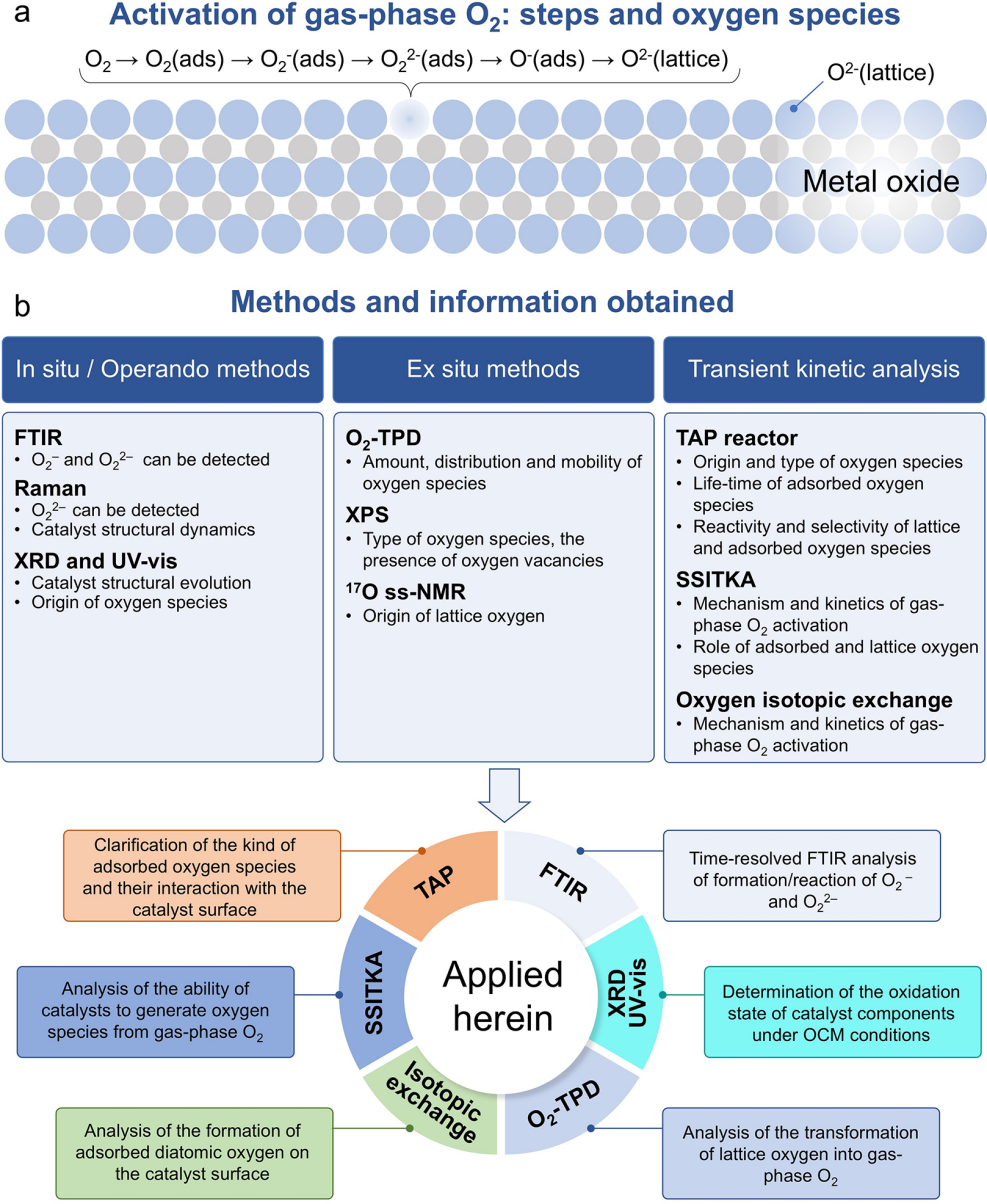
(a) Scheme showing how surface oxygen species are formed from O_2_. (b) Typical methods used for identifying surface oxygen species.

### 2.1 Mechanistic Concepts of O_2_ Activation

Oxygen temperature-programmed desorption tests are routinely used to analyze the distribution and mobility of lattice oxygen ([O]_lat_) and adsorbed oxygen species as well as to quantify their amounts.^17,18^ However, this technique does not provide insights into the mechanism of how and which oxygen species are formed from O_2_. The oxygen isotope exchange (OIE) technique overcomes this limitation, partially owing to the use of labeled oxygen, ^18^O_2_. OIE tests can be performed under isothermal or temperature-programmed conditions. Analyzing the obtained responses of ^16^O_2_, ^16^O^18^O, and ^18^O_2_ enables us to distinguish among the three possible mechanisms of the oxygen exchange reactions (Scheme 2): R_1_ (one [O]_lat_ participates), R_2_ (two [O]_lat_ participate), or R_0_ (no [O]_lat_ is involved).^19,20^ By tracking the onset temperature and relative fractions of ^16^O_2_ and ^16^O^18^O, researchers can identify whether adsorbed species or [O]_lat_ participates in the reaction with ^18^O_2_.^21^ Furthermore, OIE tests can be coupled with IR or Raman spectroscopy to directly evidence the kind of oxygen species.^22^

**Scheme 2:**
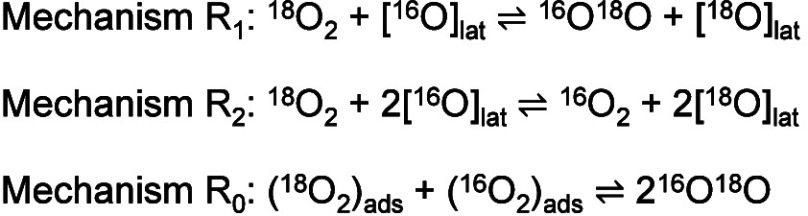
Mechanisms of Oxygen Isotopic Exchange

The temporal analysis of products (TAP) reactor operating with submillisecond resolution can indirectly provide close-to-elementary information on how O_2_ reacts with solid catalysts.^23,24^ To this end, the experimental O_2_ response obtained after the pulsing of an O_2_-containing mixture over a catalyst at the reaction temperature is subjected to a fitting procedure using different microkinetic models of O_2_ activation. The model that gives the best fit contains information about (i) the kind of surface oxygen species without considering their charge and (ii) the individual steps in their formation including the rate constants. Such an analysis performed using Na/CaO catalysts revealed that O_2_ is first reversibly adsorbed on an anion vacancy as an (O–O)_ads_ followed by a reversible dissociation to two (O)_ads_’s. The promoter accelerates the latter step.^25^ A similar model describes the activation of O_2_ over Rh/Al_2_O_3_ catalysts.^26^ The size of the Rh nanoparticles affects the ability of Rh to generate surface oxygen species. [O]_lat_ is formed directly from O_2_ over V-based catalysts.^27^

Steady-state isotopic transient kinetic analysis (SSITKA) uniquely probes the lifetime and the concentration of surface intermediates that form gas-phase products.^28–30^ This method involves switching from one reaction feed to another with the same overall composition, except that one of its components is isotopically labeled. This technique was applied to study the mechanism and the kinetics of activation of O_2_ over OCM catalysts.^31–33^ The SSITKA method enabled us to identify two distinct pools of oxygen species present in the Mn-Na_2_WO_4_/SiO_2_ system.^4,28^

### 2.2 Characterization Methods and Detected Oxygen Species in OCM

Raman and FT-IR spectroscopy provide direct evidence of adsorbed O_2_^2–^ and/or the corresponding O_2_^–^ species. For example, Lunsford et al.^34,35^ identified O_2_^2–^ on the surface of Na/La_2_O_3_, Sr/La_2_O_3_, and BaO/MgO using Raman spectroscopy. O_2_^–^ species were also observed by in situ Raman spectroscopy on the surface of rare earth metal oxides.^36^ These species were also identified on the La_2_B_2_O_7_ catalyst^37^ at 550 °C and the Mn_2_O_3_-Na_2_WO_4_/Ce_0.15_Zr_0.85_O_2_ catalyst^38^ at 680 °C using in situ FTIR spectroscopy

X-ray photoelectron spectroscopy (XPS) is a surface-sensitive technique that is widely employed to characterize and to quantify the surface composition of different catalysts including oxygen species.^39^ Typically, the O 1s peak can be deconvoluted and quantified. However, the oxygen-related signals are often broad, making it difficult to accurately discriminate between different oxygen species.^17,40^

Exclusively based on the results of single-pulse ^16^O_2_ and ^16^O_2_/^18^O_2_ pump–probe experiments in a TAP reactor, Wang et al.^41^ discussed the location of O^–^ and O_2_^2–^ or O_2_^–^ on the surface of the Na_2_WO_4_/SiO_2_ catalyst. Sourav et al.,^42^ who also conducted TAP experiments, suggested the existence of dissolved molecular oxygen released from molten Na_2_WO_4_ and [O]_lat_ associated with surface Na-WO_*x*_ sites in the Na_2_WO_4_/SiO_2_ catalyst. These statements need independent validation by other methods because the TAP experiments alone do not provide direct insight into the structure of the oxygen species.

Solid-state ^17^O nuclear magnetic resonance spectroscopy can reveal the origin of the active [O]_lat_.^43^ Using this technique for characterizing Mn_7_Si^17^O_12_-Na_2_W^17^O_4_/cristobalite, Mn_7_Si^17^O_12_/cristobalite, and Na_2_W^17^O_4_/cristobalite, Si et al.^44^ demonstrated that the [O]_lat_ of Na_2_WO_4_ does not directly participate in OCM. Instead, it modulates the [O]_lat_ of Mn_7_SiO_12_, thereby improving C_2_-selectivity.

We applied in situ XRD and operando UV–vis in a time-resolved mode to analyze the reactivity of the [O]_lat_ of Mn- or W-containing phases in the Mn-M_2_(Na, K, Rb or Cs)­WO_4_/SiO_2_ catalysts.^2,45,46^ The [O]_lat_ of MnO_*x*_ was found to show a significantly higher reactivity than the [O]_lat_ of M_2_WO_4_. This result contradicts the idea that W^6+^ is first reduced and then reoxidized by Mn^3+^ as suggested in refs 47–49 .

## 3 Type and Role of Oxygen Species Involved in Selective and Unselective Pathways

There is no doubt that surface oxygen species are needed to break the strong C–H bond(s) in CH_4_ and enable the formation of various reaction products. There are, however, uncertainties about which kind of oxygen species govern catalyst activity and, particularly, product selectivity. This is primarily caused by the limitations of state-of-the-art characterization methods to directly monitor oxygen species at high reaction temperatures. Thus, the selectivity–oxygen–speciation relationships are derived indirectly and critically reviewed below.

Wachs and co-workers have comprehensively studied the OCM reaction over the Mn-Na_2_WO_4_/SiO_2_ system using in situ XRD, UV–vis, and Raman spectroscopy (Figure 2a).^42,50,51^ The characterization studies were complemented by kinetic tests and temporal analysis of the products. The [O]_lat_’s associated with surface Na-WO_*x*_ sites were suggested to be responsible for the oxidation of CH_4_ to C_2_-hydrocarbons and surface CH_*x*_ fragments to CO. Molecular oxygen dissolved in the molten Na_2_WO_4_ phase mainly oxidizes CH_4_ to CO_2_ and is also involved in the dehydrogenation of C_2_H_6_ to C_2_H_4_. The reason that the oxidation of C_2_H_6_ stops at C_2_H_4_ and cannot proceed to CO_2_ or at least to CO, as in the case with CH_4_, needs further investigation.

**Figure 2:**
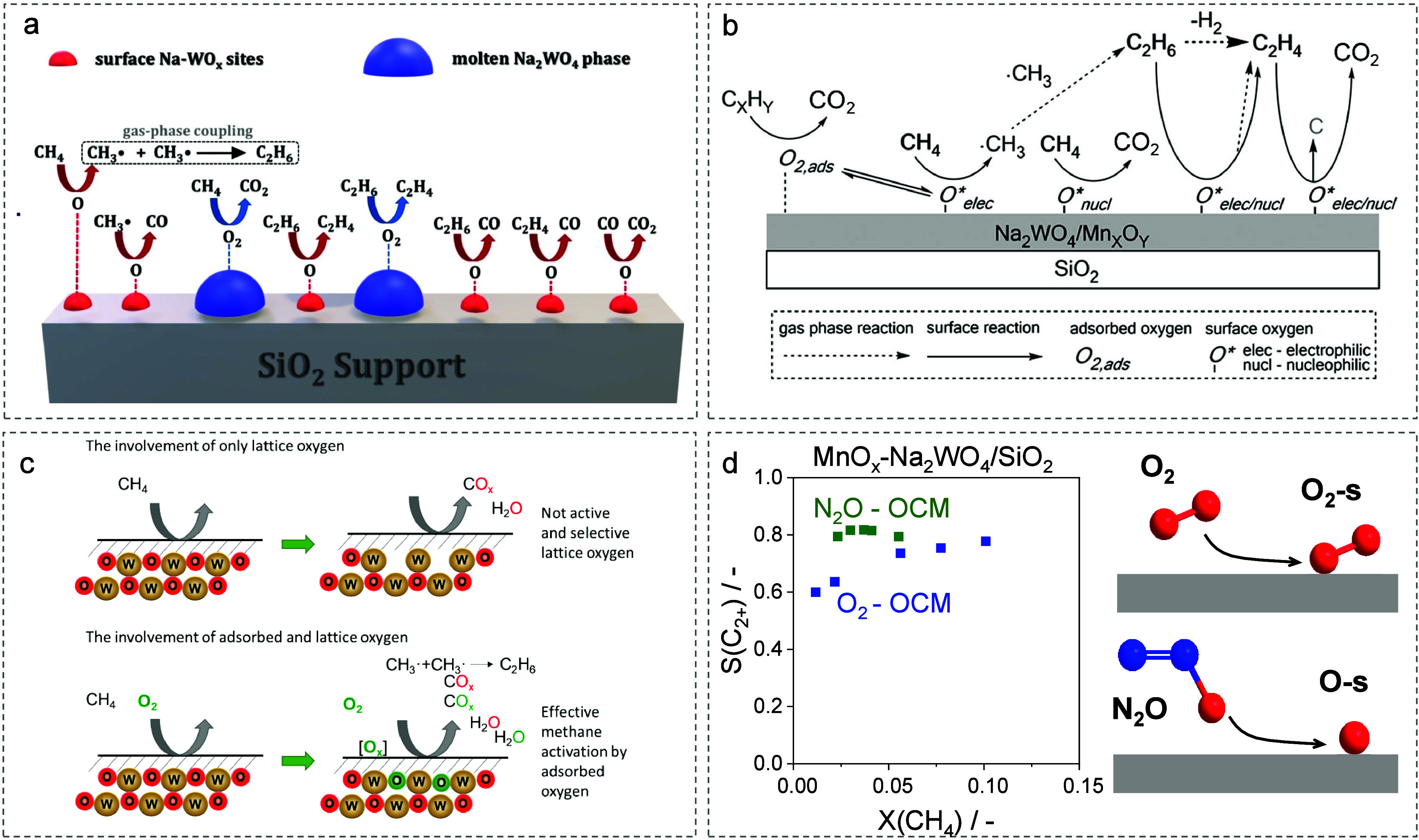
Oxygen species and their roles in the OCM. (a) Reaction steps proposed for the OCM over Na_2_WO_4_/SiO_2_. Reproduced with permission from ref 50. Copyright 2021 Wiley-VCH. (b) Proposed surface- and gas-phase reactions in the OCM over Na_2_WO_4_/Mn/SiO_2_. Reproduced with permission from ref 52. Copyright 2016 Elsevier. (c) Oxygen species involved in the formation of different products over M_2_WO_4_/SiO_2_ after pulsing of CH_4_ or CH_4_-^18^O_2_ at 800 °C. Reproduced with permission from ref 2. Copyright 2022 American Chemical Society. (d) C_2+_-selectivity versus methane conversion over Mn-Na_2_WO_4_/SiO_2_ at 800 °C and oxygen species primarily formed from O_2_ and N_2_O.

Schomäcker and co-workers also differentiated between weakly and more strongly adsorbed oxygen species or [O]_lat_ on the surface of the Mn-Na_2_WO_4_/SiO_2_ catalyst.^52,53^ Based on TAP and temperature-programmed surface reaction experiments, they proposed that (O–O)_ads_ contributes to the formation of CO_2_, whereas (O)_ads_ is involved in the formation of C_2_H_6_ (Figure 2b).

Using spatial and temporal kinetic analysis with isotopic tracers in combination with in situ XRD and operando UV–vis spectroscopy tests, our group identified that the activity of the M_2_(Na, K, Rb, Cs)­WO_4_/SiO_2_ catalysts and product selectivity are determined by the interplay between the [O]_lat_ of M_2_WO_4_ and adsorbed oxygen species formed from O_2_.^2^ Only CO and CO_2_ were formed when CH_4_ was pulsed at 800 °C (Figure 2c). C_2_-hydrocarbons were formed when CH_4_ and ^18^O_2_ were copulsed, highlighting the importance of adsorbed oxygen species formed from ^18^O_2_ in their formation. Moreover, these species show a higher reactivity than [O]_lat_ (Figure 2c). To explore the role of (O)_ads_ and/or (O–O)_ads_, we performed OCM tests using O_2_ (O_2_-OCM) and N_2_O (N_2_O-OCM) as oxidants (Figure 2d).^1,54,55^ These oxidants were chosen because O_2_ produces both (O)_ads_ and (O–O)_ads_, whereas N_2_O preferentially yields (O)_ads_ (Figure 2d). Based on the selectivity–conversion relationships for CO, CO_2_, C_2_H_4_, and C_2_H_6_ obtained in these tests, we concluded that CO and CO_2_ in O_2_-OCM are formed directly from CH_4_ and additionally through the oxidation of C_2_-hydrocarbons.^1,54,55^ Using N_2_O instead of O_2_ hindered the direct pathways, resulting in an increase in the C_2_-selectivity (Figure 2d). These results suggest that (O)_ads_ oxidizes CH_4_ to C_2_H_6_ while (O–O)_ads_ participates in the formation of CO_*x*_ in agreement with previous studies.^56,57^

## 4 Strategies for Regulating Selective Oxygen Species

As C_2_-selectivity has a great impact on the final price of produced ethylene,^16^ understanding the strategies for regulating the concentration of selective oxygen species is crucial to the commercialization of the OCM reaction. Some representative options in this direction are presented and discussed in the following sections.

### 4.1 Effects of Steam

The positive effect of steam on the formation rate of C_2_-hydrocarbons (r­(C_2_)) was reported in 2008 by Iglesia and Takanabe using the Mn-Na_2_WO_4_/SiO_2_ catalyst.^58–61^ Until 2020, this effect was thought to be exclusive to this system. Starting in that year, we published a series of studies using other catalysts including ZrO_2_/SiO_2_, PbO_*x*_/SiO_2_, K_2_MnO_4_/SiO_2_, KMnO_4_/SiO_2_, M­(Na, K, Rb or Cs)_2_WO_4_/SiO_2_, and Mn-M_2_WO_4_/support for which steam also increases C_2_-selectivity.^2,62,63^ Thus, it may be advantageous to consider the steam effect when developing the OCM catalysts. To this end, we critically analyze the concepts on this topic.

Iglesia and Takanabe^58–61^ suggested that OH radicals originating from in situ-formed H_2_O_2_ break the C–H bond in CH_4_, yielding methyl radicals which recombine to ethane. Alternatively, the group of Sinev assumed that steam helps to regenerate catalytically active sites.^64^ These concepts do not consider whether and how steam influences the formation of CO_*x*_ and C_2_-hydrocarbons to explain the steam-mediated increase in the C_2_-selectivity.

To bridge this fundamental gap, we investigated the OCM reaction over Mn-Na_2_WO_4_/SiO_2_, MnO_*x*_/SiO_2_, and Na_2_WO_4_/SiO_2_ under alternating steam-free (dry cycle) and steam-containing (wet cycle) feeds. The enhancing steam effect on the rate of CH_4_ consumption was found to be reversible and its strength depends on the composition of the catalyst (Figure 3a). Reversible and irreversible steam-mediated effects on the C_2_-selectivity were identified (Figure 3b). The latter was attributed to the redispersion of large MnO_*x*_ particles, hindering CO_*x*_ formation. The steam effect on C_2_-selectivity increases as the reaction temperature decreases due to the different changes in the rates of CH_4_ conversion to C_2_-hydrocarbons, CO, and CO_2_ (Figure 3c,d). Moreover, steam lowers the activation energy of the formation of C_2_-hydrocarbons more strongly (Figure 3e), which is crucial for achieving high C_2_-selectivity at low temperatures. As steam increased the rates of CH_4_ conversion to C_2_H_6_, C_2_H_4_, CO_2_, and CO to different extents,^63,65^ OH radicals cannot be the only reason for enhancing C_2_-hydrocarbon formation as suggested in refs 58–61 .

**Figure 3:**
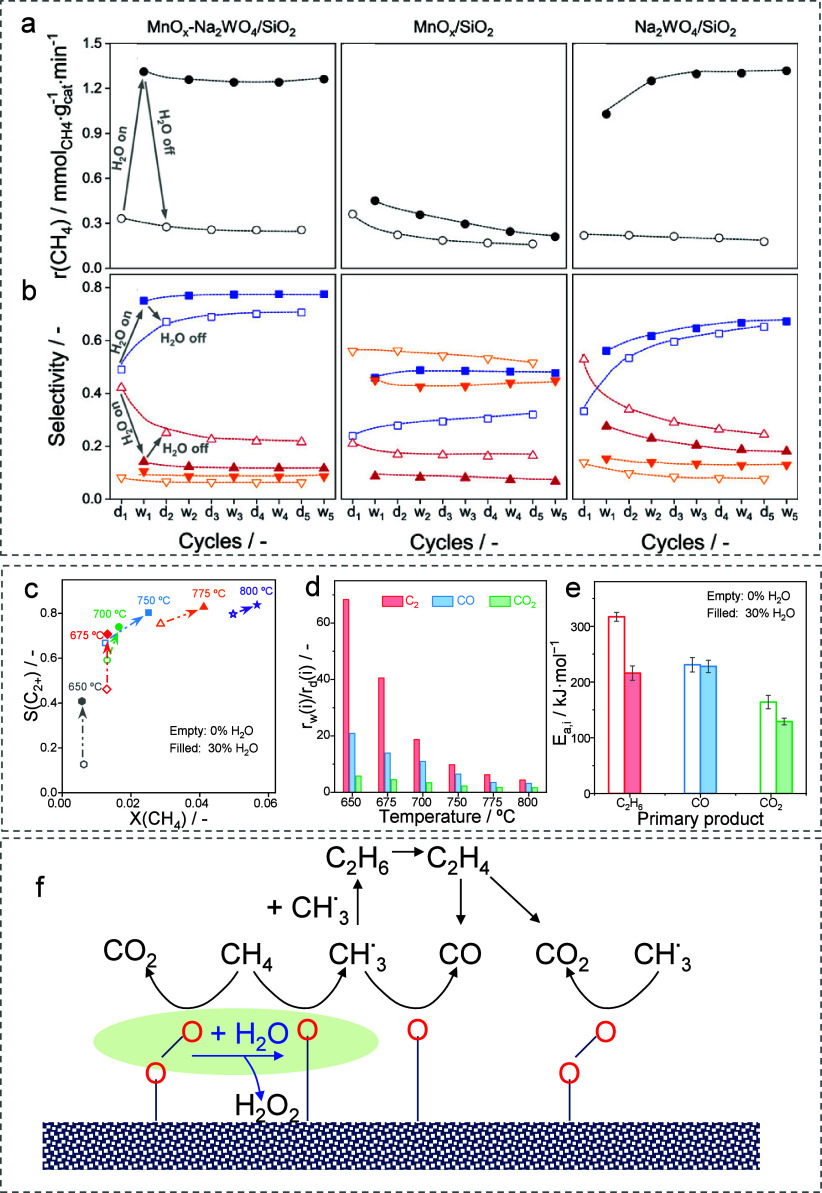
(a) Overall rate of methane conversion and (b) C_2_ selectivity (blue solid and open squares), CO_2_ (red solid and open triangles), and CO (yellow solid and open triangles) over Mn-Na_2_WO_4_/SiO_2_, MnO_*x*_/SiO_2_, and Na_2_WO_4_/SiO_2_ in dry and wet cycles. Reproduced with permission from ref 63. Copyright 2020 American Chemical Society. (c) Effect of temperature on C_2+_ selectivity without (empty symbols) and with (filled symbols) steam. (d) Ratios of CH_4_ conversion rates into C_2_-hydrocarbons (red bars), CO (blue bars), and CO_2_ (green bars) with steam to those without steam (rw­(i)/rd­(i)). (e) Apparent activation energies of C_2_H_6_, CO, and CO_2_ formation without (empty bars) and with (filled bars) steam. Reproduced with permission from ref 65. Copyright 2023 Elsevier. (f) Scheme illustrating the mechanism of the steam effect on the reaction pathways in OCM.

Clear evidence of the heterogeneous nature of the steam effect was provided by TAP experiments under conditions free of radical reactions using ^18^O_2_-CH_4_ mixtures with and without steam. Larger amounts of CO_*x*_ were formed in the presence of steam. Moreover, the presence of ^16^O stemming from the catalyst and ^18^O originating from ^18^O_2_ in carbon oxides suggests that both [O]_lat_ and adsorbed oxygen species participate in their formation. The C^16^O/C^18^O ratio increased, whereas the C^16^O_2_/(C^18^O^16^O + C^18^O_2_) ratio decreased in the presence of steam. These results together with the steam effect on the CO_2_ selectivity allowed us to suggest that (O–O)_ads_ participates in the formation of CO_2_. Steam accelerates the dissociation of (O–O)_ads_ to (O)_ads_ and H_2_O_2_ (Figure 3f). (O)_ads_ shows a higher C_2_-selectivity than [O]_lat_. On this basis, we improved the OCM performance of different catalysts by cofed steam.^62,63,66,67^

### 4.2 Chemical Looping Approach and Addition of Li_2_CO_3_

As (O–O)_ads_ is involved in the oxidation of CH_4_ to CO_2_, performing the OCM reaction in a chemical looping mode (CL-OCM) is an attractive approach for increasing C_2_-selectivity because CH_4_ and O_2_ are alternatingly fed to the reactor for product formation using [O]_lat_ and the reoxidation of the reduced catalysts, respectively. However, CL-OCM suffers from low productivity due to the limited amount of reactive [O]_lat_. Inspired by the steam effect on O_2_-OCM, we enhanced the productivity of the Mn-Na_2_WO_4_/SiO_2_ catalysts in CL-OCM using cofed steam without compromising C_2_-selectivity (Figure 4a).^46^ This improvement was explained by the reaction of H_2_O with (O–O)_ads_ formed by the recombination of two [O]_lat_’s. This reaction yields [O]_ads_ with higher reactivity than that of [O]_lat_. The best-performing catalyst was also durable in a series of 50 cycles (Figure 4b).

**Figure 4:**
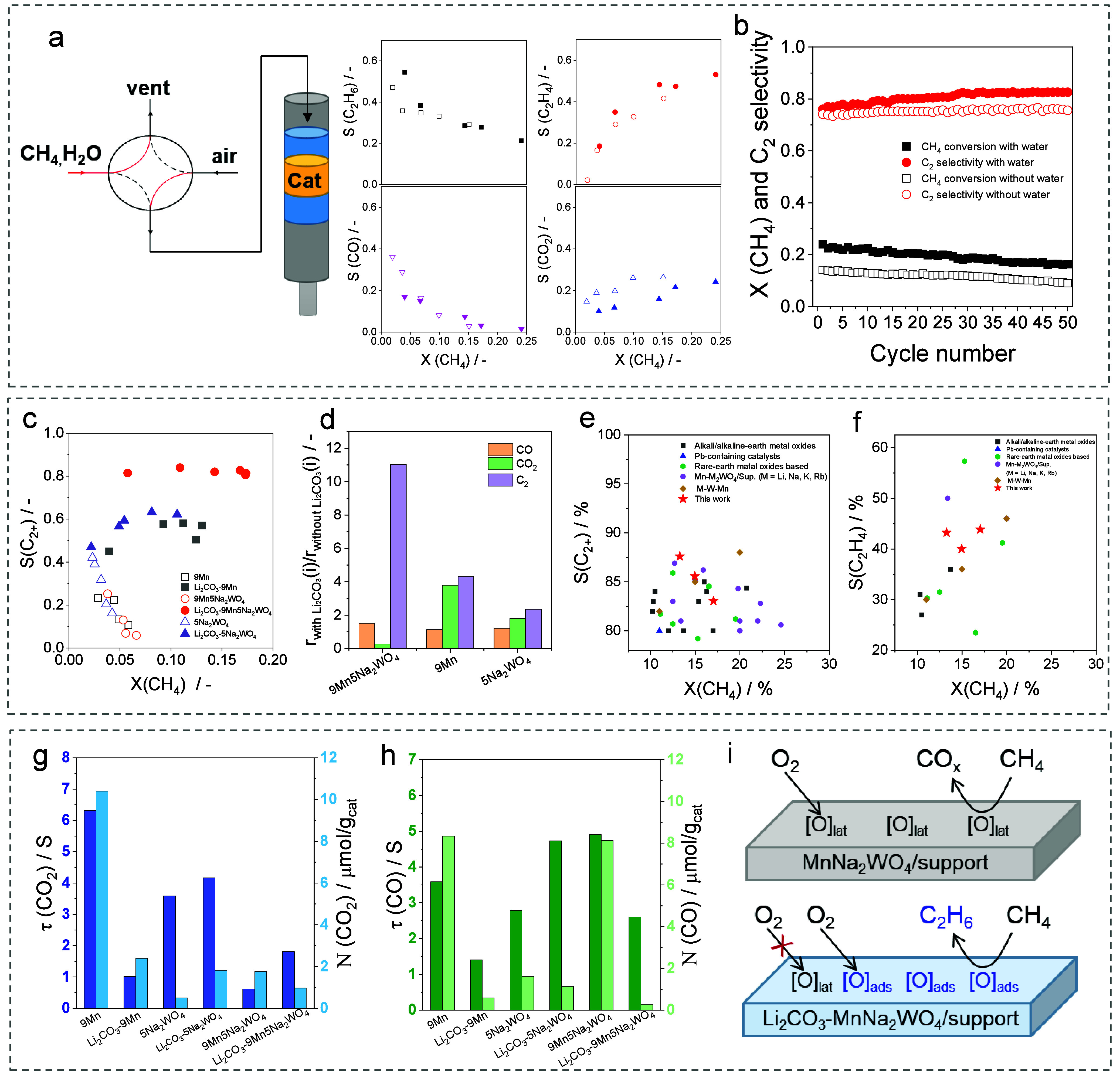
(a) Selectivity–conversion relationships of different products and (b) CH_4_ conversion and C_2_-selectivity in 50 cycles in CL-OCM over Mn-Na_2_WO_4_/SiO_2_ with (filled symbols) and without (empty symbols) steam. Reproduced with permission from ref 46. Copyright 2023 Elsevier. (c) C_2+_-selectivity versus methane conversion over Mn/Siral70, Na_2_WO_4_/Siral70, and Mn-Na_2_WO_4_/Siral70 with (closed symbols) and without (open symbols) Li_2_CO_3_. (d) The ratios of the rate of CH_4_ conversion to an individual product with added Li_2_CO_3_ to that without Li_2_CO_3_. Selectivity–conversion values for (e) C_2+_-hydrocarbons and (f) ethylene obtained in our study^4^ and previous O_2_-OCM studies. Lifetime and concentration of surface intermediates yielding (g) CO_2_ and (h) CO calculated from the ^13^CH_4_-^16^O_2_-SSITKA tests. (i) A scheme of the enhancing effect of Li_2_CO_3_ on product selectivity. Reproduced with permission from ref 4. Copyright 2025 American Chemical Society.

Very recently, Li_2_CO_3_-coated mixed rare earth metal oxides were reported as a new class of CL-OCM catalysts.^68^ The Li_2_CO_3_-LaPrO_3+*x*_ catalyst achieved a 30% yield of C_2_-hydrocarbons with C_2_-selectivity below 80%. The high performance was related to the presence of a molten layer of Li_2_CO_3_ on the catalyst surface. This layer is important for the formation of H_2_O_2_, which is converted to OH radicals promoting CH_4_ activation. Beside the surface layer, the Li-induced oxygen vacancies in the (W)-Mg_6_MnO_8_ lattice bind [O]_lat_ more strongly after reacting with O_2_. This oxygen species is less active for the formation of CO_*x*_.^69,70^

The addition of Li_2_CO_3_ to various Mn-Na_2_WO_4_/support catalysts also improves their CL-OCM performance.^71^ C_2_-selectivity reached 89% (about 60% C_2_H_4_ selectivity) at a methane conversion of 19% when steam was cofed with CH_4_. As no Li_2_CO_3_ phase was found in the spent catalysts, the presence of a molten carbonate layer cannot be considered relevant to the enhancement effect. Therefore, lithium must play a decisive role in this enhancement.

In contrast to the Li_2_CO_3_-LaPrO_3+*x*_ system, physically mixing Li_2_CO_3_ or LiNO_3_ with Mn-Na_2_WO_4_/support catalysts improved their performance in O_2_-OCM (Figure 4c).^4^ MnO_*x*_ and Na_2_WO_4_ in these catalysts act synergistically (Figure 4d). C_2_-selectivity of 90.5 or 87.6% was obtained at 7.2 or 13.3% CH_4_ conversion. These values are remarkable when compared with those of different OCM catalysts from the database with about 5100 data points (Figure 4e,f).^72^ Li_2_CO_3_ or LiNO_3_ was found to react irreversibly with the catalyst support, yielding highly crystalline materials with low specific surface area, which is advantageous for hindering heterogeneous CH_4_ oxidation to CO_*x*_. The selectivity improvements were rationalized by the SSITKA and TAP tests. The Li_2_CO_3_-induced phase modifications affect both the concentration (N­(i)) and the lifetime (τ­(i)) of surface intermediates leading to CO_2_ and CO (Figure 4g,h). These structural modifications modulate the ability of the catalysts to produce selective oxygen species from O_2_ (Figure 4i).

### 4.3 Roles of MnO_*x*_ and Alkali Metal in Mn-M_2_WO_4_/SiO_2_

Mn-Na_2_WO_4_/SiO_2_ is one of the most promising OCM catalysts. Despite numerous studies, the roles of M_2_WO_4_ (M = Na, K, Rb, or Cs) and MnO_*x*_ are still controversially discussed. As Na_2_WO_4_ is present in the molten phase under OCM conditions, some researchers suggested that the oxygen released through the interaction of Mn^3+^ with the melt plays a significant role.^73,74^ In contrast, Wachs and co-workers proposed that the [O]_lat_ associated with isolated Na-WO_*x*_ oxidizes CH_4_ to C_2_H_6_.^42,50,51^ However, Rb_2_WO_4_/SiO_2_ and K_2_WO_4/_SiO_2_ catalysts exhibit higher or similar C_2_-selectivity than Na_2_WO_4/_SiO_2_, although the former tungstates do not melt under the reaction conditions.^75,76^

Using spatially resolved and temporal kinetic analysis with isotopic tracers, we thoroughly investigated the OCM reaction over the (Mn)-M_2_WO_4_/SiO_2_ (M = Na, K, Rb, or Cs) catalysts.^45,2^ Their ability to generate adsorbed oxygen species from O_2_ was identified as a key activity-governing descriptor.^2^ This catalyst property was quantified by the amount of ^16^O^18^O formed in temperature-programmed OIE experiments by using a feed with ^18^O_2_. This amount increases with an increase in the electronegativity of the alkali metal in M_2_WO_4_ (Figure 5a). There is also a correlation between the rates of CH_4_ conversion to C_2_H_6_ and CO_2_ with this amount. No dependence could be established for the rate of CH_4_ conversion to CO. These results indicate that adsorbed oxygen species contribute to the formation of C_2_H_6_ and CO_2_, while CO is formed with the participation of [O]_lat_ (Figure 5b). As the rates of conversion of CH_4_ to C_2_H_6_ and CO_2_ increase with the electronegativity of the alkali metal (Figure 5c), the electronic properties of the catalysts govern the activation of O_2_ to yield adsorbed oxygen species.

**Figure 5:**
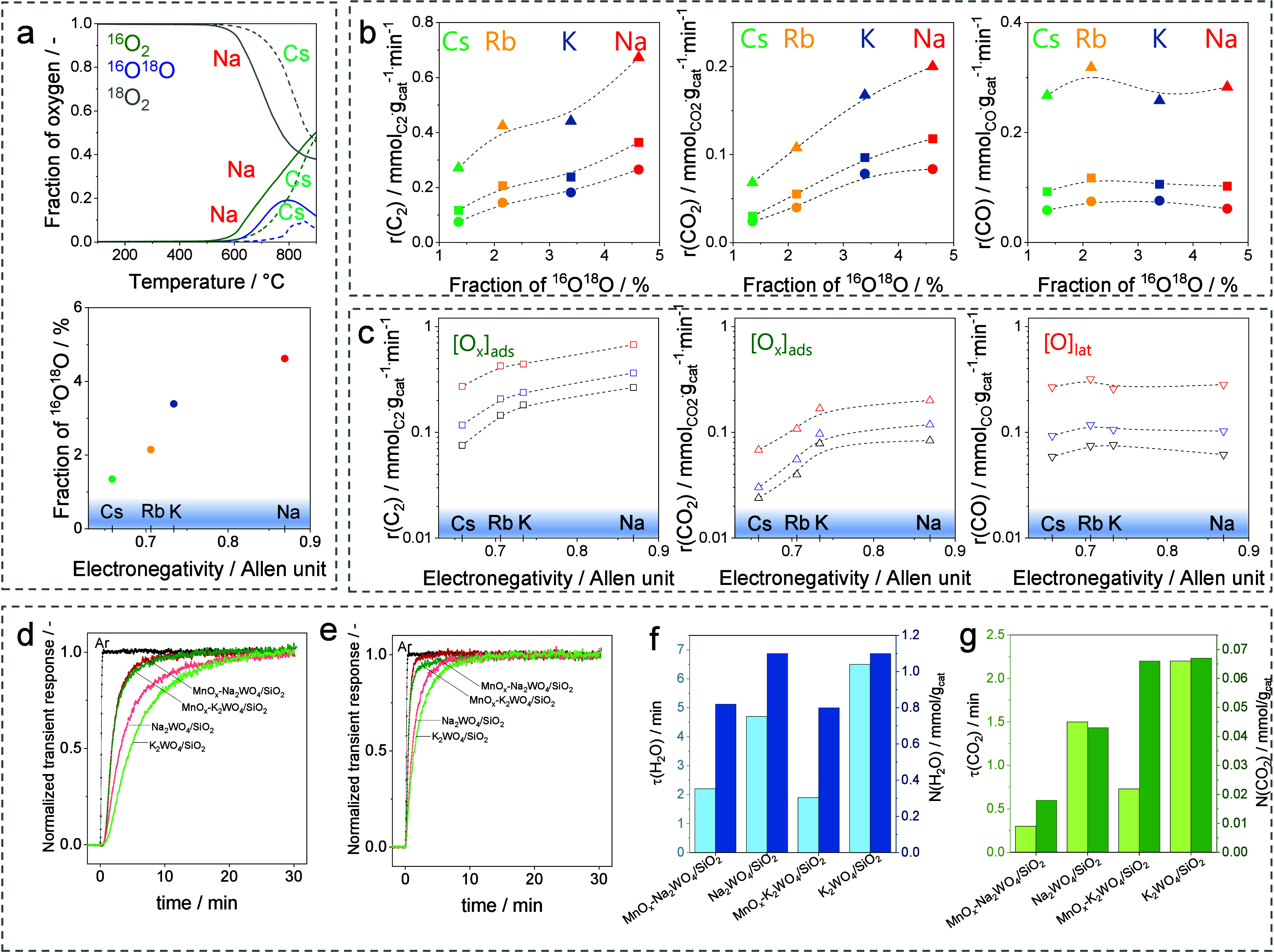
(a) Temperature profiles of differently labeled oxygen detected during temperature-programmed OIE experiments with M_2_WO_4_/SiO_2_ (M = Na, K, Rb, Cs) and the fraction of ^16^O^18^O versus the electronegativity of M. The formation rates of C_2_-hydrocarbons and CO and CO_2_ over M_2_WO_4_/SiO_2_ at different CH_4_/O_2_ ratios (circle 12:1, square 8:1, triangle 4:1) versus (b) the fraction of ^16^O^18^O from (a) or versus (c) the electronegativity of M. Reproduced with permission from ref 2. Copyright 2022 American Chemical Society. Normalized transient responses of (d) Ar and H_2_^18^O or (e) Ar, C^16^O^18^O, and C^18^O_2_ after switching from a CH_4_/^16^O_2_/He = 30/4/66 feed to a CH_4_/^18^O_2_/Ar/He = 30/4/1/65 feed at 775 °C. (f) τ­(H_2_O) (left bars) and N­(H_2_O) (right bars). (g) τ­(CO_2_) (left bars) and N­(CO_2_) (right bars). Reproduced with permission from ref 45. Copyright 2023 Elsevier.

The role of MnO_*x*_ in the (Mn)-M_2_(Na or K)­WO_4_/SiO_2_ catalysts was investigated by the SSITKA method.^45^ After switching from the ^16^O_2_/CH_4_ feed to the ^18^O_2_/CH_4_ feed, the normalized temporal profiles of H_2_^18^O and of C^18^O^16^O plus C^18^O_2_ responses of the Mn-Na_2_WO_4_/SiO_2_ and Mn-K_2_WO_4_/SiO_2_ catalysts reached their steady-state values faster than those of the Mn-free counterparts (Figure 5d,e). According to SSITKA theory,^23,30,77^ these responses were used to calculate τ­(i) and N­(i) of surface intermediates leading to H_2_O and CO_2_. The τ­(H_2_O) value of the Mn-containing catalysts is lower compared with that of the Mn-free catalysts, while the catalysts do not obviously differ in N­(H_2_O) (Figure 5f). These results suggest that Mn does not increase the number of active sites but rather enhances their intrinsic reactivity. Importantly, the τ­(H_2_O) value of all catalysts is larger than the τ­(CO_2_) value (Figure 5f,g), suggesting that the rate-limiting step cannot be the cleavage of the C–H bond(s) in CH_4_. The regeneration of active sites through water formation should control the catalyst activity.

## 5 N_2_O Is a Promising Oxidant for the Oxidative Coupling of Methane

### 5.1 Gd_2_O_3_ Promoted by BaO Is a Highly Efficient Catalyst System for N_2_O-OCM

Although N_2_O is more expensive than air for large-scale applications, it has also been investigated as an oxidant in OCM to understand how different oxygen species influence product selectivity.^7,8,11^ This is because N_2_O primarily yields (O)_ads_, whereas O_2_ initially generates (O–O)_ads_.^6,9,78^ The use of N_2_O instead of O_2_ has been shown to lead to reduced methane conversions but increased C_2_-selectivity. From a mechanistic point of view, N_2_O suppresses the direct oxidation of CH_4_ to CO_2_, enhancing the C_2_-selectivity.^1,56,57^ Nevertheless, selectivity values of above 80% have been reported at CH_4_ conversion degrees of below 10%. In addition, typical N_2_O-OCM catalysts operate above 750 °C.

Very recently, our group discovered a new class of N_2_O-OCM catalysts based on Gd_2_O_3_ promoted with BaO (BaGd), which afford 90% C_2_-selectivity at industrially relevant CH_4_ conversion at 650 and 700 °C.^3,54^ Both the selectivity and activity depend on the promoter concentration and reach their highest values at 10 wt % Ba loading (0.1BaGd). The impact of the achieved performance is illustrated in Figure 6a. The 0.1BaGd catalyst showed a selectivity of 90% at 11% CH_4_ conversion at 700 °C, whereas most reported catalysts required above 750 °C but performed less selectively. CH_4_ conversion could be increased to 15% without losing the high selectivity upon optimizing the feed composition (Figure 6b).

**Figure 6:**
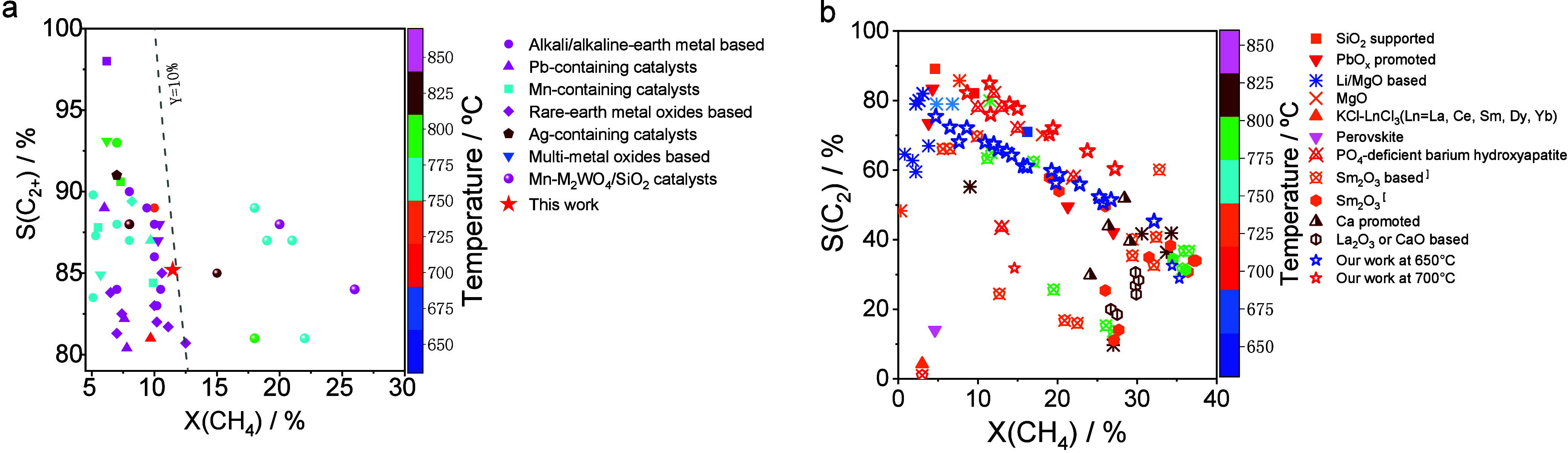
Selectivity–conversion values of C_2+_- or C_2_-hydrocarbons obtained over 0.1BaGd and various previous catalysts tested in (a) O_2_-OCM and (b) N_2_O-OCM.

Mechanistic insights into the positive role of the promoter and N_2_O were elucidated by analyzing the selectivity–conversion relationships of CO, CO_2_, C_2_H_4_, and C_2_H_6_ obtained through varying the space velocity.^3,54^ CO and C_2_H_6_ were identified as the only products formed directly from CH_4_ in both N_2_O-OCM and O_2_-OCM. CO_2_ is also a primary product in N_2_O-OCM over bare Gd_2_O_3_ and 0.01BaGd. Increasing the Ba loading strongly decreases the impact of the direct unselective pathways, in favor of the formation of C_2_H_6_. The kinetic origins of the enhancing effect of the promoter are provided in the following section.

### 5.2 Kinetic Descriptors for Efficient Production of C_2_-Hydrocarbons in N_2_O-OCM

Pump–probe experiments in the TAP reactor using N_2_O and CH_4_ proved that adsorbed oxygen species formed from N_2_O break the C–H bonds in CH_4_.^3,54^ This conclusion was made based on the fact that the intensity of the O_2_ response in the N_2_O pulse decreased sharply when CH_4_ entered the reactor (Figure 7a). When the time delay between the N_2_O and CH_4_ pulses was increased, methane conversion decreased due to the recombination of adsorbed oxygen species to yield O_2_. To qualitatively estimate the effect of Ba loading on the latter reaction, we used the time of maximum intensity (*t*_max_) of the response of the O_2_ in the N_2_O pulse. The *t*_max_ value increases as the loading increases due to the hindrance of the recombination of adsorbed oxygen species. This promoter effect is detrimental to the rate of CO_*x*_ formation in N_2_O-OCM over both the BaGd catalysts and the Na- or Sr-promoted Gd_2_O_3_ catalysts.^3,54^

**Figure 7:**
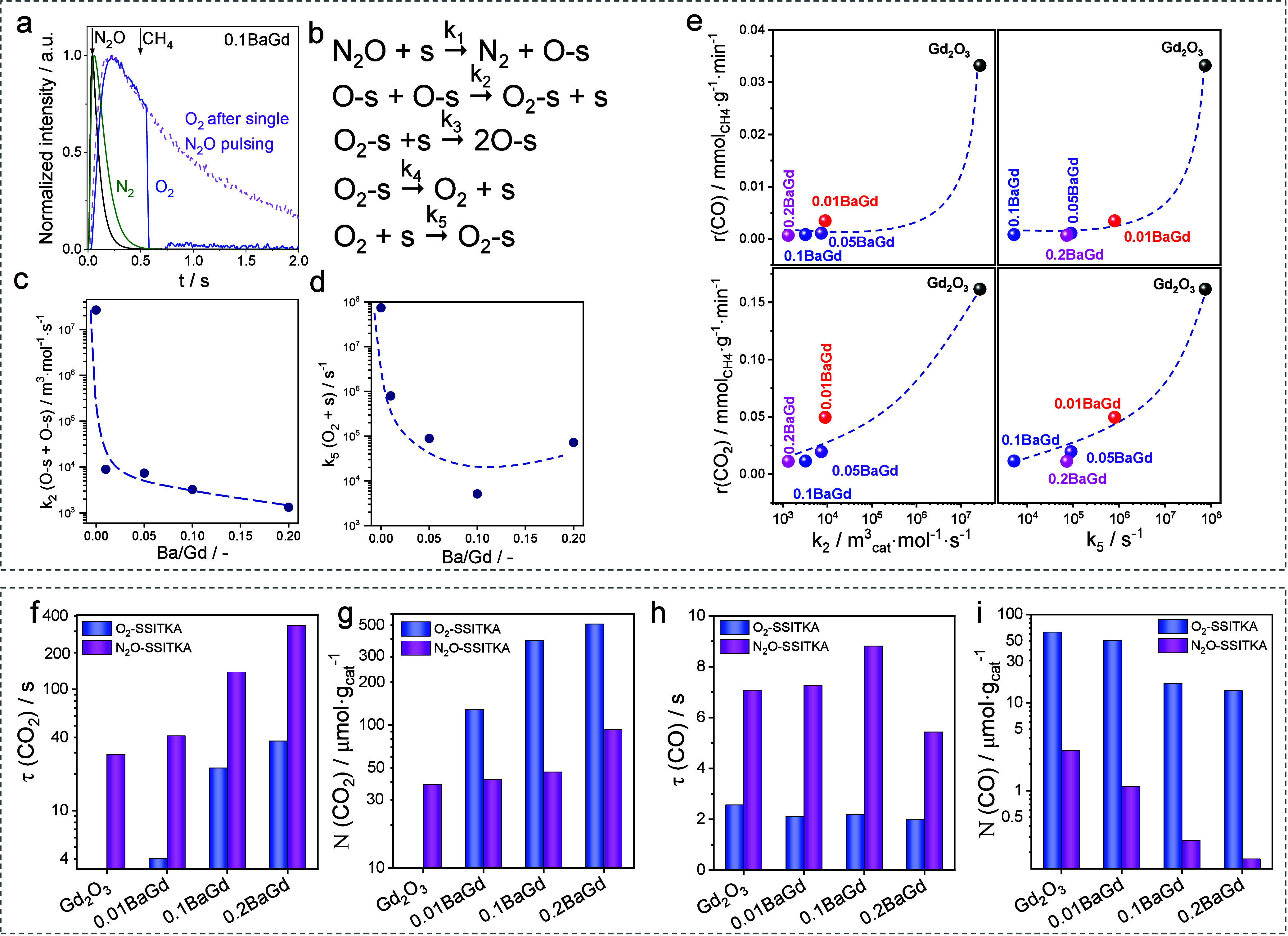
(a) Height-normalized responses of N_2_O and O_2_ after N_2_O-CH_4_ pump–probe tests with a time delay of 0.5 s over 0.1BaGd. Reproduced with permission from ref 3. Copyright 2024 Wiley-VCH. (b) The best microkinetic model of N_2_O decomposition was derived through discrimination among three different models. The rate constant of (c) O-s recombination (*k*_2_) and (d) O_2_ adsorption (*k*_5_) versus the loading of Ba in Ba/Gd_2_O_3_. (e) Rates of methane conversion to CO and CO_2_ in N_2_O-OCM over Ba/Gd_2_O_3_ versus *k*_2_ and *k*_5_. (f, h) Lifetime and (g, i) concentration of intermediates leading to CO_2_ and CO over Ba-promoted Gd_2_O_3_ catalysts as determined in the ^13^CH_4_-O_2_-SSITKA and ^13^CH_4_-N_2_O-SSITKA tests. Reproduced with permission from ref 54. Copyright 2025 American Chemical Society.

Insights into the mechanism of N_2_O decomposition were derived by fitting the experimental responses of N_2_O, N_2_, and O_2_ obtained after pulsing N_2_O in the TAP reactor to three microkinetic models differing in the pathways leading to the reaction products.^3,54^ The model that provided the best fit is shown in Figure 7b. (O)_ads_ is directly formed from N_2_O irreversibly, followed by the recombination of two such species to an adsorbed (O–O)_ads_ which desorbs as O_2_. Using the obtained rate constants of the individual reaction steps in Figure 7b, we found a strong negative effect of the Ba loading in BaGd on the *k*_2_ and *k*_5_ values (Figure 7c,d). Thus, the promoter hinders the formation of (O–O)_ads_. These species should be involved in the formation of CO_*x*_ as can be concluded from the correlation between the formation rates of these products and the *k*_2_ and *k*_5_ values in Figure 7e.

To rationalize the above effects, we performed SSITKA tests with ^12^CH_4_/O_2_/He = 9/1/90 and ^13^CH_4_/O_2_/Ne/He = 9/1/2/88 (O_2_-SSITKA) as well as ^12^CH_4_/N_2_O/He = 9/2/89 and ^13^CH_4_/N_2_O/Ne/He = 9/2/2/87 (N_2_O-SSITKA) mixtures. The τ­(i) and N­(i) of the surface intermediates leading to CO and CO_2_ were determined from these tests (Figure 7f–i). N­(CO_2_) is significantly lower in N_2_O-OCM than in O_2_-OCM probably due to the lower concentration of (O–O)_ads_. The kind of such intermediates should depend on the oxidant used, as reflected by the different τ­(CO_2_) values, which are higher in N_2_O-OCM. For both oxidants, N­(CO_2_) and τ­(CO_2_) increase with increasing Ba loading, which suggests that the promoter plays a pivotal role in the formation of CO_2_. Conversely, the promoter has a negative effect on N­(CO), which is more pronounced in N_2_O-OCM (Figure 7i). However, based on the τ­(CO) values in Figure 7h, the kind of surface intermediates does not depend on Ba loading but is affected by the oxidant used.

In summary, the derived fundamentals regarding the kind of oxygen species and their influence on C_2_-selectivity can be used to develop catalysts that operate selectively in O_2_-OCM. As O_2_ primarily yields (O–O)_ads_, efficient catalysts should convert this species to (O)_ads_ as quickly as possible to hamper the formation of CO_*x*_.

## 6 Summary and Outlook

To achieve a breakthrough in the development of large-scale processes based on the OCM, it is essential to understand how to improve C_2_-selectivity through catalyst design or optimizing reaction conditions. In this Account, we have demonstrated the potential of transient and steady-state mechanistic studies with isotopic tracers including their (micro)-kinetic analysis as well as sophisticated time-resolved in situ/operando catalyst characterization to elucidate the role of the kind of oxygen species in controlling product selectivity. The valuable mechanistic insights we have derived enabled us to improve the selectivity–conversion trade-off of the well-known Mn-Na_2_WO_4_ system and to develop a promising system based on Gd_2_O_3_ promoted with Ba to N_2_O-OCM. Our achievements demonstrate the potential of studies focusing on the relationships between the (micro)­kinetics/mechanism of oxidant activation and catalytic performance not only in the OCM but also in other selective oxidation reactions.

To conclude this Account, we identified the following research areas relating to the development of OCM catalysts that may find applications in industry.

(i) Although alkali/alkali earth metal oxides have been often used as a promoter for improving the C_2_-selectivity of various catalysts,^10–15^ an unexpected breakthrough was recently achieved using large amounts of Li_2_CO_3_/LiNO_3_ to improve the performance of rare earth metal oxides^68^ or Mn-Na_2_WO_4_-based catalysts.^4,71^ In this view, it is worth applying this approach to other OCM catalysts. The role of these additives was, however, discussed controversially, thus highlighting the need for further detailed mechanistic and kinetic studies. It is especially important to understand if the promoting effect is related to Li, e.g., its small size. In the latter case, other additives can also be used. However, ensuring high catalyst onstream stability can be a critical challenge when working with molten compounds due to their instability at high temperatures.
(ii) The potential of using reaction feeds with steam for improving C_2_-selectivity should be further investigated for catalysts different from the well-studied Mn-Na_2_WO_4_ system. Given that steam’s enhancing effect grows stronger as the reaction temperature drops, similar studies should also be conducted below 750 °C. CL-OCM with steam is also an underexplored approach in view of recent achievements.^46^
(iii) Given the heterogeneous–homogeneous nature of the OCM reaction, a deeper understanding of the interplay between catalyzed and gas-phase reactions is needed to control product selectivity. This can be achieved through comprehensive kinetic modeling.^79^ Specifically, synchrotron vacuum ultraviolet photoionization mass spectrometry allows for distinguishing between surface oxygen-mediated activation and gas-phase chain reactions.^80,81^

(iv) A significant drawback in the understating of OCM catalysts for their tailored development is the limited applicability of state-of-the-art characterization methods for unambiguously establishing structure–activity–selectivity relationships and identifying selective and unselective oxygen species under operando conditions due to very high reaction temperatures. Combining IR/Raman spectroscopy with SSITKA seems to be a promising approach, as these methods can complement each other by providing information about the type, number, and concentration of surface intermediates of gas-phase reaction products.
(v) As the concentration of the feed components and the reaction products changes along the catalyst bed, catalyst composition can also change accordingly. Conducting catalytic and characterization tests in a spatially resolved manner will certainly extend our knowledge of structure–activity–selectivity relationships. Thus, further developments of in situ/operando cells resembling catalytic reactors are needed.
